# Operative versus non-operative management of rib fractures in flail chest after cardiopulmonary resuscitation manoeuvres

**DOI:** 10.1093/icvts/ivac023

**Published:** 2022-02-03

**Authors:** Patrick Dorn, Selina Pfister, Simone Oberhaensli, Konstantinos Gioutsos, Matthias Haenggi, Gregor J Kocher

**Affiliations:** 1 Department of Thoracic Surgery, Inselspital, Bern University Hospital, University of Bern, Bern, Switzerland; 2 Interfaculty Bioinformatics Unit and SIB Swiss Institute of Bioinformatics, University of Bern, Bern, Switzerland; 3 Department of Intensive Care Medicine, Inselspital, Bern University Hospital, University of Bern, Bern, Switzerland

**Keywords:** Rib fracture, Flail chest, Rib stabilization, Chest wall stabilization, Cardiopulmonary resuscitation

## Abstract

**OBJECTIVES:**

Blunt chest trauma after mechanical resuscitation manoeuvres appears to have a significant impact on the often complicated course. Due to a lack of data in the literature, the purpose of this study was to investigate the feasibility and immediate outcome of chest wall stabilization for flail chest in this vulnerable patient population.

**METHODS:**

We retrospectively reviewed the medical records of patients after cardiopulmonary resuscitation between January 2014 and December 2018 who were diagnosed with flail chest. We attempted to compare patients after surgery with those after conservative treatment.

**RESULTS:**

Of a total of 56 patients with blunt chest trauma after mechanical resuscitation and after coronary angiography, 25 were diagnosed with flail chest. After the exclusion of 2 patients because of an initial decision to palliate, 13 patients after surgical stabilization could be compared with 10 patients after conservative therapy. Although there was no significant difference in the total duration of ventilatory support, there was a significant advantage when the time after stabilization to extubation was compared with the duration of ventilation in the conservative group. The presence of pulmonary contusion, poor Glasgow Coma Scale score or the development of pneumonia negatively affected the outcome, but additional sternal fracture did not.

**CONCLUSIONS:**

Surgical stabilization for chest wall instability is well tolerated even by this vulnerable patient population. Our results should be used for further randomized controlled approaches. It is necessary to evaluate the situation with all parameters in an interdisciplinary manner and to decide on a possible surgical therapy at an early stage if possible.

**Figure:**
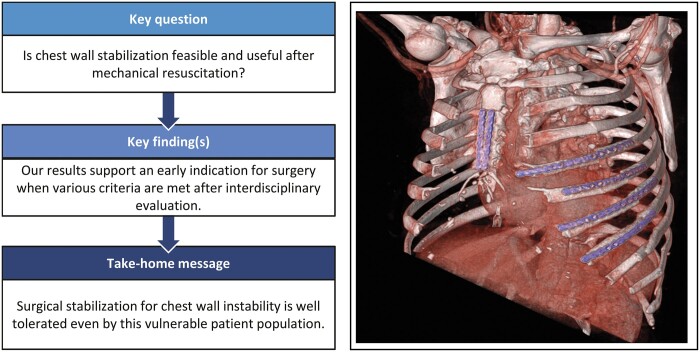

## INTRODUCTION

Rib fractures are common and are diagnosed in up to 39% of patients with blunt chest trauma [1, 2]. They are associated with increased pulmonary morbidity (e.g. pneumonia) in 17–77% and with a mortality rate of ∼10%, which increases with patient age and the number of fractured ribs [3]. Rib fractures also commonly occur after chest compressions during cardiopulmonary resuscitation (CPR) for cardiac arrest [4, 5]. Chest wall instability seems to have a negative impact on the overall clinical outcome and also on the immediate rehabilitation course after CPR. The deterioration of respiratory mechanics due to rib fractures, changes in cardiopulmonary physiology and also the impairment of weaning from mechanical ventilation have been suggested as causes [6].

Historically, rib fractures and chest wall instability have been treated conservatively, with appropriate analgesia, respiratory physiotherapy and early mobilization. The development of continuous epidural administration of anaesthetic agents via catheters, as well as the use of endobronchial lavage options, has further optimized this approach and is standard of care for most rib fractures and also for many resulting chest wall instabilities. In parallel, however, thanks to the impressive development of surgical rib stabilization, a considerable treatment option has emerged that has now led to a paradigm shift in the treatment of rib fractures. In certain situations, rib fractures and chest wall instability following blunt chest trauma can be treated more aggressively by stabilizing the ribs and thoracic wall with promising results [7, 8]. Patients who were surgically stabilized had lower mortality rates, shorter intensive care unit (ICU) and hospital length of stay, shorter duration of mechanical ventilation, lower tracheotomy rates and fewer pneumonias compared with patients who were not surgically treated [9]. Age, Glasgow Coma Scale (GCS) score, ventilatory support, number of comorbidities and status after CPR manoeuvres have been identified as risk factors affecting mortality in patients with unstable chest. These factors have already been integrated into risk scores to identify candidates for surgical treatment who are likely to benefit most from this treatment option due to an overall better probability of survival. The authors of one study concluded that, according to their scoring system, patients with low to intermediate risk as determined by the above factors may be good surgical candidates [10]. Rib stabilization in the remaining patients with a high score or the presence of more than one of the above factors should not be performed because the outcome is determined by the underlying disease and not by the instability of the chest.

The situation of patients with an unstable chest wall after CPR is very complex because of the combination of trauma and underlying disease. According to the current state of the literature, there is still a lack of information on the impact of surgical stabilization of the ribs on the potential rehabilitation benefit in these patients, who are not only in a critical condition due to the current event, but usually also suffer from other cardiac and non-cardiac comorbidities. To date, there are no standardized guidelines for the management of rib fractures secondary to CPR manoeuvres, and according to the current literature, there are few case reports of surgical therapy as a treatment option in this patient population [11, 12]. The purpose of this study is to evaluate the feasibility of chest wall stabilization in these vulnerable patients and to perform a comparative retrospective analysis regarding the already demonstrated benefit of this operation for the treatment of flail chest in a different situation (e.g. for blunt chest trauma without cardiac event).

## PATIENTS AND METHODS

### Patients and study design

We retrospectively searched for patients having undergone successful CPR leading to flail chest between January 2014 and December 2018 at Inselspital, University Hospital Bern, Switzerland. In this patient population, coronary angiography was performed to clarify the suspected cardiac or medical cause of the cardiac arrest. The indication for emergency coronary angiography after resuscitation with return of spontaneous circulation was given by our colleagues of interventional cardiology. Electrocardiogram changes of ST-elevated myocardial infarction were clear reasons, but depending on the situation also, for example, ventricular fibrillation as initial heart rhythm and instability, or other rhythms with signs of cardiac ischaemia. The first registered cardiac rhythm after CPR manoeuvre and the intervention during coronary angiography are listed in Supplementary Material, Table S6. The diagnosis of flail chest was made by a thoracic surgeon after suspicion was raised by the attending physicians in the ICU in patients with no obvious contraindication from a neurological point of view (GCS ≥ 9). For clinical assessment, respiratory support from the ventilator was suspended, and the diagnosis was based on paradoxical movements and palpation of clearly separated wall segments. In addition, the presence of computed tomography of at least the chest supporting the diagnosis of flail chest after resuscitation with a radiological correlate was mandatory. The minimum length of stay in the ICU of 5 days or longer should exclude a premature decision to discontinue therapy and also allow the necessary time to observe the weaning process. Exclusion criteria were: CPR for non-cardiac/non-medical reasons, shorter ICU stay (<5 days) and pre-existing disorders of consciousness. Only failure to wean from mechanical ventilation was considered as a surgical indication. Other indications, such as optimization of the pain situation, resulted in exclusion from the study cohort. Neurological assessment of patients was performed using a sedation-adapted GCS score, which was determined by different ICU staff during daily patient rounds to avoid misinterpretation and to ensure continuous assessment throughout the stay. The GCS score obtained on the day of treatment option decision was included in the analysis. In summary, failure to wean patients with a radiologically and clinically confirmed flail chest without severe neurological deficits was the reason for the interdisciplinary evaluation and decision to proceed. The final decision to perform surgical stabilization was then made solely by the experienced thoracic surgeon involved and, according to our analysis, was not influenced by either extended anticoagulation or the other factors mentioned.

The primary endpoint when comparing groups (surgically treated vs non-surgically treated patients) was total time spent on ventilatory support. Secondary endpoints were surgically induced and non-surgically induced complications, such as bleeding and infection in the chest wall and intrathoracic, other bleeding due to anticoagulation (e.g. gastrointestinal bleeding), pneumonia and neurological deterioration and need for tracheotomy.

### Ethics approval

This single-centre retrospective analysis was approved by the Ethics Committee Bern, Switzerland, on 27 August 2019 (KEK Bern 2019-01122). The study was conducted in accordance with the Helsinki Declaration as revised in 2013.

### Surgical procedure

For surgical rib or sternal stabilization, either the MatrixRIB™ Fixation System (Depuy Synthes, Johnson & Johnson AG, Zuchwil, Switzerland) or the RibFix Blu™ Thoracic Fixation System (Zimmer Biomet, Warsaw, IN, USA) was used.

**Figure 1:**
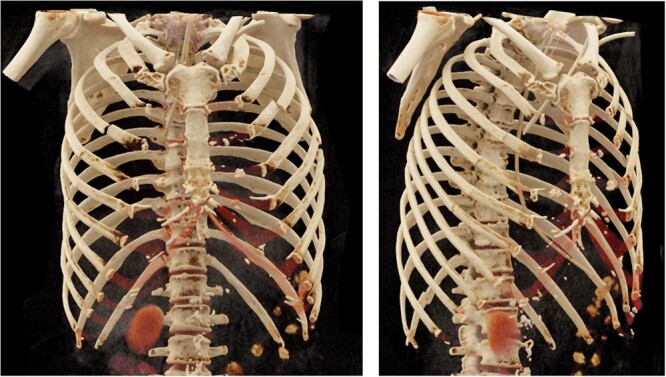
Three-dimensional reconstruction of post-cardiopulmonary resuscitation computed tomography scan chest: rib fractures 2–7 (5–7 multifragmented) and 12 right, 2–7 left, transverse sternal body fracture, clavicle shaft fracture (between mid and lateral third of clavicle, not visible in reconstruction).

**Figure 2:**
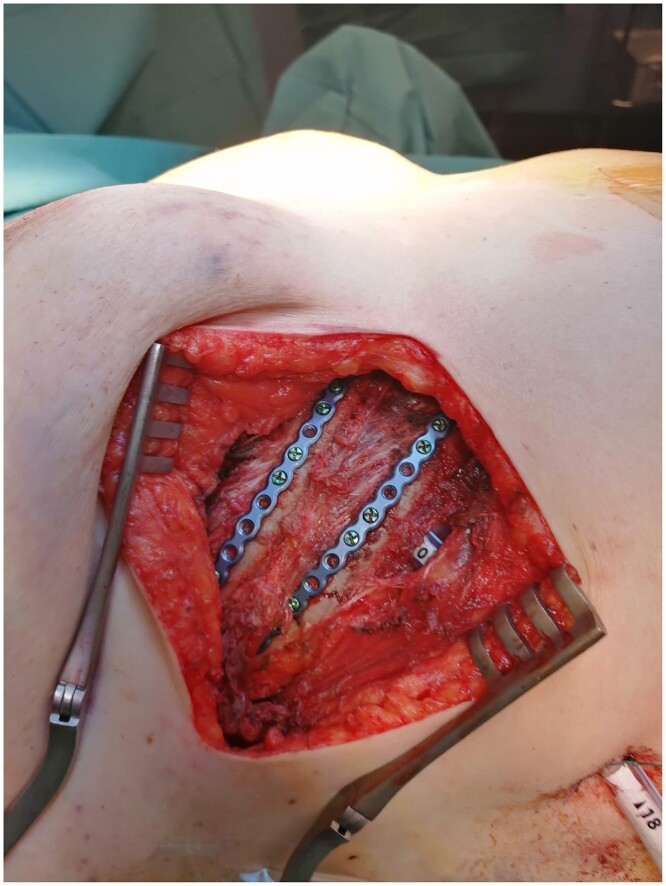
Submammary incision for rib stabilization 3–6 on right side (RibFix Blu™, only plates on ribs 4 and 5 visible).

**Figure 3:**
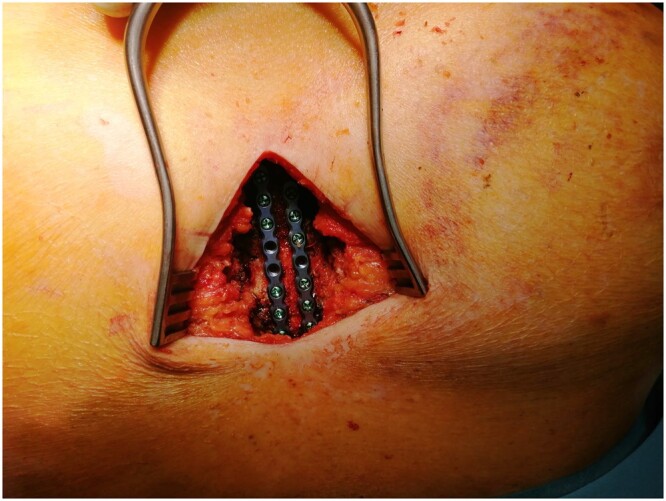
Sternal plating (2 × 8-hole-plates RibFix Blu™).

**Figure 4:**
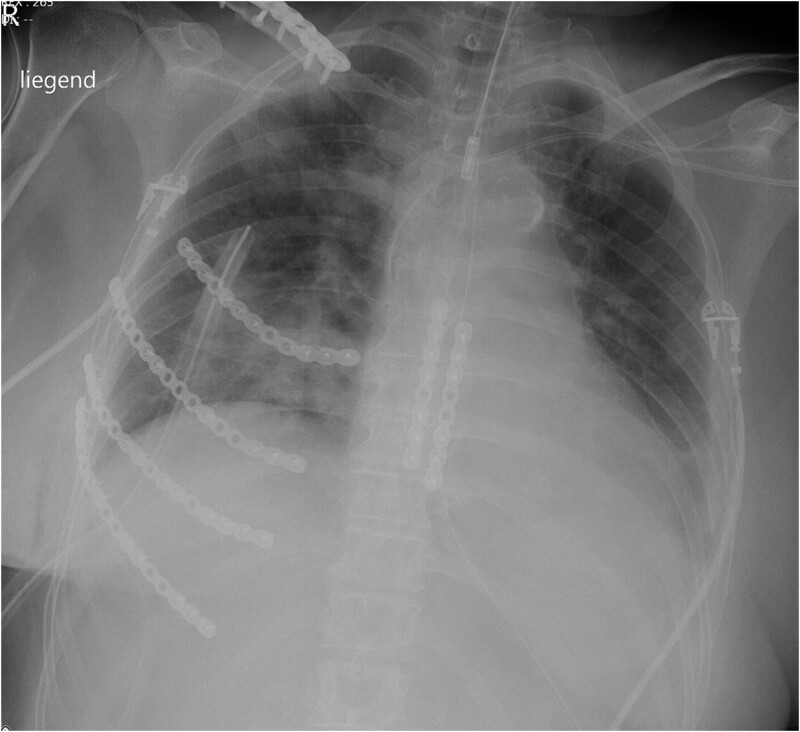
Postoperative X-ray after sternal stabilization, stabilization of ribs 3–6 on the right side and right clavicle.

The perioperative positioning of the patient was determined depending on the fractures to be treated. Patients were positioned in either lateral, semi-lateral or supine position. In our experience, lateral and ventral rib fractures remain the best to fix. Posterior paravertebral rib fractures should be stabilized only in exceptional cases and did not occur in this study population. Mechanical ventilation with a double-lumen tube was necessary if a simultaneous thoracoscopy was planned, for example, to evacuate a residual haemothorax at the same time. Otherwise, in our opinion, single-lumen intubation is sufficient, if necessary in combination with occasional apnoea phases to avoid lung damage during stabilization. The number of incisions and the number of ribs to be fixed were determined based on several criteria: extent of the fracture pattern, accessibility of the fractures (important especially for multifragment fractures) and need for stabilization of the sternum. The primary goal is to achieve optimal stability with as little soft tissue damage and osteosynthesis material as possible (Figs 1–4 as an example of the indication, further planning and surgical treatment of a flail chest after CPR manoeuvres).

### Data collection and statistical analysis

Patient characteristics and potential risk factors for a complicated or prolonged course of ventilatory support during the ICU stay were assessed using data from our hospital’s electronic medical record. Based on literature and experience, we hypothesized that conservative treatment of the flail chest, the patient’s neurological condition, the presence of pulmonary contusion after CPR and the extent of the fracture pattern might influence the duration of ventilatory support. Therefore, we investigated these and other parameters.

Quantitative data are described by mean ± standard deviation or median and range (Supplementary Material, Table S5). Differences between groups with respect to length of stay on the ventilator were assessed with unpaired Student’s *t*-tests [data are approximately normally distributed, ‘see Quantile Quantile (QQ) plots’ in Supplementary Material (Supplementary Material, Fig. S1)]. Differences between groups in the incidence of complications and the frequency of tracheotomies were analysed with Fisher’s exact test. Data from patients who died during hospitalization were excluded from analyses when time on the ventilator was used. This is because the ventilator time for these patients does not reflect the time from admission to the time the ventilator could be removed, but rather the time from admission to death and therefore is not comparable to the data for the other patients.

Multivariable linear regression models were fitted to the data to examine the relationship between length of stay on the ventilator and the other variables studied. Backward selection with the Akaike information criterion as an optimization parameter was applied to reduce model complexity and identify relevant predictors. We used the likelihood ratio test after the backward selection procedure to test and fine tune the outcome of backwards selection. All statistical analyses were performed using R (http://www.R-project.org/). The code and detailed results of all tests performed can be downloaded from https://github.com/IBUunibe/Dorn_2022_ICVTS.

## RESULTS

Considering the above criteria ‘CPR’, ‘coronary angiography after CPR’, ‘chest wall injury’ and ‘length of stay on ventilator ≥5 days’, our search in the electronic medical records of Inselspital, University Hospital Bern, Switzerland, yielded a total of 56 patients. Of these 56 patients, 28 did not meet the ‘flail chest’ criterion, and 3 patients refused consent for data collection and use. Of the remaining 25 patients, 13 patients were treated by surgical stabilization of the chest wall (Sx group) and 10 patients were treated conservatively (No-Sx group). We did not include 2 patients into the conservatively treated group for analysis because the family decided to convert to palliative care with comfort therapy during hospitalization. These 2 patients died on Days 5 and 6, respectively, after CPR. The 2 comparison groups were very similar and comparable with respect to many important characteristics such as age, fracture pattern and GCS score (Supplementary Material, Tables S5 and S6).

**Figure 5:**
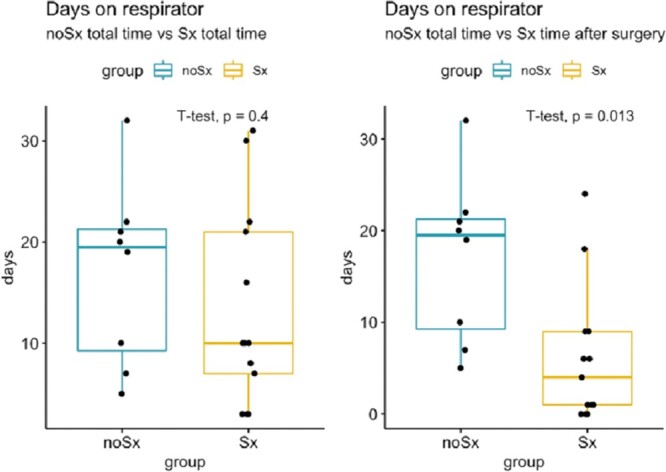
Comparison between the 2 groups (Sx and no-Sx) regarding time of respirator dependence.

The primary outcome of our study is the required duration of ventilatory support with successful extubation as the endpoint and expression of a successful weaning process. During the ICU stay corresponding to the observation period for this study, in addition to the palliative patients, 2 patients in the No-Sx group died on Days 6 and 13, respectively, after CPR. In contrast, no patient in the Sx group died during the observation period (Supplementary Material, Table S6). When comparing both groups (No-Sx group versus Sx group) in terms of total duration of ventilatory support, there was no statistically significant difference [Fig. 5, left; mean Sx group: 13.3 days, mean No-Sx group: 17 days, 95% confidence interval (CI) −5.28 to 12.51, *P* = 0.4].

As the median of the Sx group is considerably smaller than the median of the No-Sx group (Fig. 5, left), we investigated more in detail the time spent on the ventilator before and after the stabilization in the Sx group. We noticed that 8 of the patients had to wait more than 3 days until the stabilization was performed (range 4–30 days, Supplementary Material, Fig. S7), whereas many of the patients could be taken from the respirator immediately or within few days after the surgery. We therefore compared the ventilation time after chest wall stabilization to extubation in the Sx group with the total ventilation time in the conservatively treated group and found that it was significantly shorter (Fig. 1, right; mean Sx group: 6 days, mean No-Sx group: 17 days, 95% CI 2.67–19.16, *P* = 0.013).

Regarding the secondary endpoints, none of the complications were found to be significantly overrepresented in either group. A comparison of the 2 groups in terms of analgesic consumption could not be performed because of the heterogeneity of analgesics and the different indications. No complications occurred during the perioperative course in the Sx group, and there were no postoperative complications such as wound infections, foreign body infections or bleeding under anticoagulation during the observation period in the ICU either. Furthermore, no revision surgical procedures were necessary.

The number of tracheotomies performed was not significantly different in the 2 groups either regardless of the therapeutic approach (Fisher’s exact test, *P* = 0.68). The indication for tracheotomy was the occurrence of neurological deterioration (3/9 vs 2/6) or insufficient weaning progress (6/9 vs 4/6) during the further course of hospitalization after interdisciplinary assessment of the indication for surgery.

In addition to the primary and secondary endpoints defined for the study, we wanted to examine the relationship between the variable ‘length of stay on the ventilator’ and the other variables, we assessed from the medical records.

To this end, we used multiple linear regression combined with backward selection (see Patients and methods) to test whether length of stay on the ventilator could be predicted by 1 or more of the explanatory variables we examined.

Using data from both groups (*n* = 21), we found that the explanatory variables ‘tracheotomy’ (*β* = 10.1, *P* = 0.005) and ‘pulmonary contusion’ (*β* = 6.7, *P* = 0.047) significantly predicted the response variable ‘total time on ventilator’ (*R*^2^ adjusted = 0.47, *F*(2,18) = 10.02, *P* = 0.001) (Supplementary Material, Table S1 and Fig. S2).

Next, we performed multiple linear regression with the same explanatory variables as before, but for the Sx group, we used the time spent on the ventilator after stabilization instead of total time as the response variable.

We found that the explanatory variables group (*β* = −10.0, *P* = 0.002), tracheotomy (*β* = 7.6, *P* = 0.012) and pulmonary contusion (*β* = 7.4, *P* = 0.015) significantly predicted the length of stay on the ventilator (*R*^2^ adjusted = 0.67, *F*(3,17) = 14.57, *P* < 0.000) (Supplementary Material, Table S2 and Fig. S3).

From these analyses, we conclude that tracheostomy is the most significant indicator of prolonged stay on the ventilator, whereas pulmonary contusion is an additional but less significant indicator.

As a next step, we analysed the data of the No-Sx and Sx group separately to investigate if we can identify indicators of longer duration of ventilation specific for the treatment approach.

### Possible indicators of severe outcomes in the surgery group (Sx group)

The median length of stay on the ventilator was 10 days in the Sx group (Supplementary Material, Fig. S7). Considering the characteristics of the patients whose length of stay in the ICU was below this median (*n* = 5), the absence of pronounced pulmonary contusions and good GCS scores of 14 or higher are striking. Of note in these 5 patients is a relatively high proportion of sternal fractures, but this does not appear to have a negative impact on outcome. The fracture pattern and the number of stabilized fractures in the total number of fractures show no obvious influence on the duration of ventilation.

In the remaining patients (*n* = 8) who were ventilator dependent for a prolonged period (>10 days), there were retrospectively more cerebral abnormalities on imaging due to unclear neurological status (5/8 vs 1/5). There was also a significant incidence of tracheotomy after stabilization in this subgroup (8/8 vs 1/5).

Neither the full model nor the model resulting from variable selection containing 6 predictors (age, sex, sternal fracture, pulmonary contusion, pneumonia and neurology) showed a significant effect of any of the explanatory variables (*R*^2^ adjusted = 0.33, *F*(6,6) = 1.9, *P* < 0.45).

**Figure 6:**
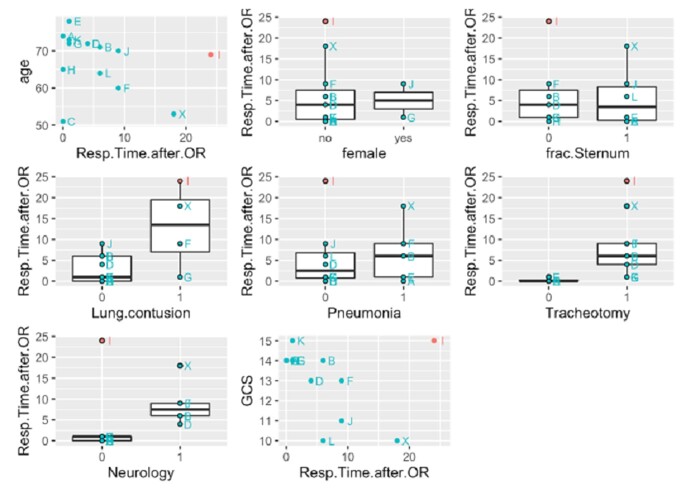
Data from Sx group including patient I (highlighted in red).

We then looked more closely at the data from the Sx group and found that 1 patient stood out (see multiplot Fig. 6, below): patient I (highlighted in red) not only suffered pulmonary contusion after mechanical resuscitation but also spent an additional 24 days on the ventilator after chest wall stabilization was performed, with unremarkable neurology and a GCS of 15, and also received a tracheotomy during the course of ventilation. However, a unique feature of this patient is that thoracic wall stabilization was performed simultaneously with a necessary triple aortocoronary bypass surgery. The sternotomy, which was reclosed with cerclages, probably had an additional negative impact on the restricted respiratory mechanics. However, postoperative delirium that developed after this long combined procedure determined long-term ventilation in this patient because of the deterioration of the neurological situation and the necessary drug therapy.

We therefore decided to exclude patient I and performed model fitting again as described in the Patients and methods section. This procedure yielded a model with 3 explanatory variables (*R*^2^ adjusted = 0.79, *F*(3,8) = 15.36, *P* < 0.002), ‘tracheotomy’ (*β* = 4.0, *P* = 0.042), ‘GCS’ (*β* = −1.9, *P* = 0.003) and ‘pneumonia’ (*β* = 4.2, *P* = 0.018), all of which are statistically significant (Supplementary Material, Table S3 and Fig. S4).

Based on these results, we hypothesize that, in addition to tracheostomy, low GCS and pneumonia may be indicators of longer duration of ventilation after stabilization.

### Possible indicators for severe courses in the No-Sx group

To investigate possible indicators in this group, we excluded from the analysis the 2 patients who died during their stay in the ICU. Five of the remaining 8 patients had a length of stay on the ventilator of more than 17.5 days (Supplementary Material, Fig. S6). These patients had a higher incidence of pneumonia (3/5 vs 0/3), a lower mean GCS (11.4 vs 14) and a higher rate of tracheotomy (4/5 vs 0/3). With only 8 data points, regression analysis and variable selection are challenging, so some explanatory variables were excluded from the full model, either due to clinical considerations or results of previous analyses. We found that ‘tracheotomy’ (*β* = 13, *P* = 0.026) is the only explanatory variable that can predict the length of stay on ventilator (*R*^2^ adjusted = 0.52, *F*(1,6) = 6.2, *P* < 0.026) (Supplementary Material, Table S4 and Fig. S5).

## DISCUSSION

Rib fractures frequently occur during CPR manoeuvres. Surgical stabilization of the unstable chest after trauma appears to improve patient outcomes by reducing pneumonia rates, shortening critical care time and also overall hospital stay, and generally optimizing treatment costs [2, 3, 9]. The efficacy of stabilization after CPR has not been studied in this fragile patient population and remains controversial. The main priority of treatment in the patient cohort of this study is, of course, the cardiac event and its rehabilitation, as well as the neurological sequelae and their progression. Most likely, this fact affects the timing of assessment of the unstable chest at prolonged weaning. In any case, the median time of 6 days (range 3–30 days) after blunt chest trauma to surgery is later than the 72-h time interval recommended in the general trauma guidelines [13]. Our observation that weaning from the ventilator is statistically significantly shorter when comparing the time on the ventilator measured only from the time of stabilization to extubation in the surgical group with the total time on the ventilator in the conservative group (No-Sx group) also indicates a possible influence of this criterion. Although our comparison between surgical and conservative therapy of flail chest after CPR manoeuvres does not show any obvious advantages, such as a lower pneumonia rate after stabilization, this intervention also seems to be surprisingly well tolerated by this severely ill patient group. From our results, we deduce that the indication for stabilization should be discussed as early as possible. An interdisciplinary decision, at least between intensivist and thoracic surgeon, is essential to assess the relevance of the flail chest based on the clinical course of the patient so far and on its mechanical properties.

We did not expect the high number of tracheotomies, which was even higher in the Sx group than in the No-Sx group. Unfortunately, the indication for tracheotomy was not made in an interdisciplinary manner and could sometimes be an expression of an almost too hasty expectation of long-term ventilation in this group of patients, influenced by hospital economic aspects, perhaps also a sign of lack of confidence in stabilization after mechanical resuscitation. However, the fact that tracheostomy was found to have a significant effect on the ventilation duration in our study can also be retrospectively interpreted as a quality characteristic for the indication for this procedure. Since in the Sx group either prolonged weaning, e.g. due to developed pneumonia, or unexpected neurological deterioration in the further course after chest wall stabilization led to tracheostomy, accurate assessment of neurological status as well as early detection of ‘non-mechanical’ respiratory limitations is enormously important for the interdisciplinary decision of stabilization. Regression analysis confirms the negative effect of lung contusion on ventilatory outcome that we suspected and that has been described in other studies [14]. The GCS as an expression of neurological status also proves to be a relevant indicator in our Sx group after CPR, as e.g. Zehr *et al.* [10] have already shown in their study with a presumably much healthier population.

### Limitations

The major limitations of this study are certainly the retrospective design as well as the small number of patients and thus the limited statistical power. The latter is mainly due to our strict inclusion criteria. However, in our opinion, these also help to enable a comparison of the inherently quite different medical histories in the 2 groups.

The absence of complications directly caused by surgery and of deaths during the observation period of ICU stay indicates that this procedure is well tolerated even by this highly susceptible group of patients. Our results and observations should therefore prompt investigation of chest wall stabilization for flail chest after CPR manoeuvres in a randomized controlled setting.

## CONCLUSION

Blunt chest trauma with rib fractures or even an unstable chest is a common injury after mechanical resuscitation. For this reason, the latter injury should be considered in the differential diagnosis, at the latest when weaning from ventilatory support makes no or insufficient progress. Surgical stabilization of an unstable chest wall is well tolerated, even by this vulnerable patient population that has already suffered a major cardiac event and is often receiving concomitant antiplatelet therapy/anticoagulation. Although some of the demonstrated benefits of stabilization are not clearly expressed in our study, our results should be used for further randomized controlled approaches. For this purpose, it is necessary to evaluate the situation with all mentioned parameters in an interdisciplinary manner and to decide on a possible surgical therapy as early as possible.

## SUPPLEMENTARY MATERIAL

Supplementary material is available at *ICVTS* online.

## Supplementary Material

ivac023_Supplementary_Data

## Notes

Presented at the 35th Annual Meeting of the European Association for Cardio-Thoracic Surgery, Barcelona, Spain, 13–16 October 2021.

## ABBREVIATIONS

CI: Confidence interval
CPR: Cardiopulmonary resuscitation
GCS: Glasgow Coma Scale
ICU: Intensive care unit
