# Supplementary material for: Operative versus non-operative management of rib fractures in flail chest after cardiopulmonary resuscitation manoeuvres
Source: Interact Cardiovasc Thorac Surg. 2022 Feb 3;34(5):768–74. doi: 10.1093/icvts/ivac023 (PMC9070522; doi:10.1093/icvts/ivac023)
Supplement: ivac023_Supplementary_Data [file ivac023_supplementary_data.docx]

**Operative versus non-operative management of rib fractures in flail chest after cardiopulmonary resuscitation**

Supplementary Figures and Tables

**
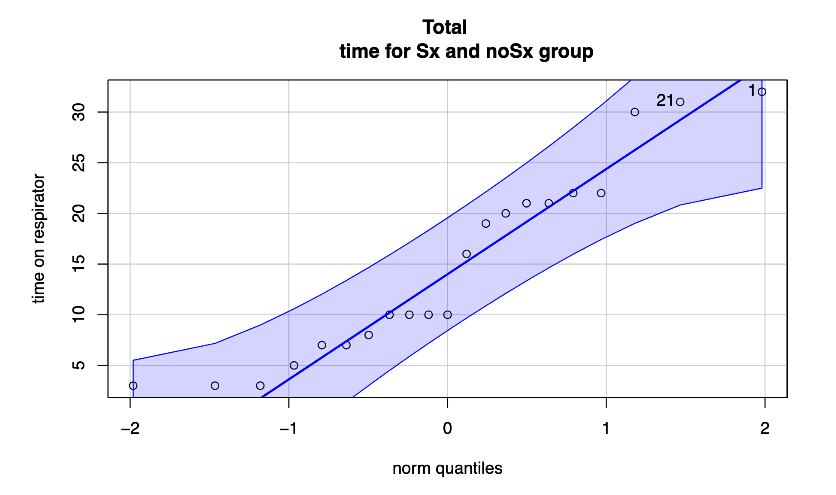

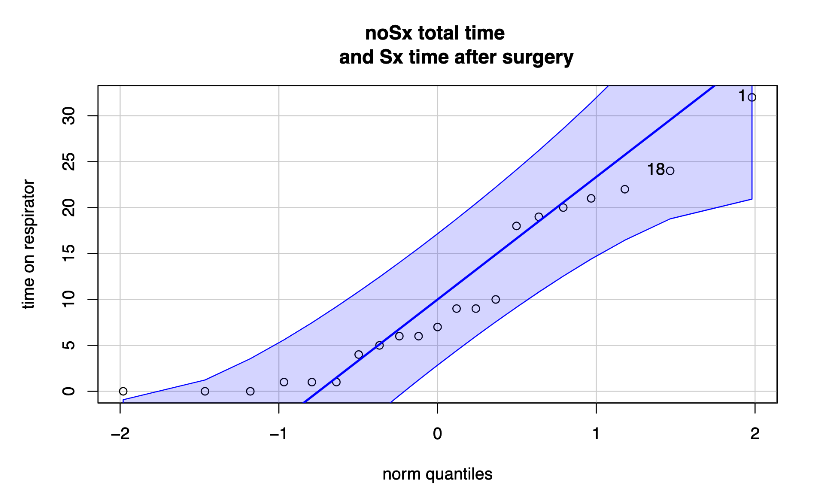
**

Supplementary Figure S1: Quantile-Quantile (QQ) plot of total time on ventilator for both groups (upper figure) and time after stabilization for Sx group and total time for No-Sx group (lower figure), showing that data are approximately normally distributed
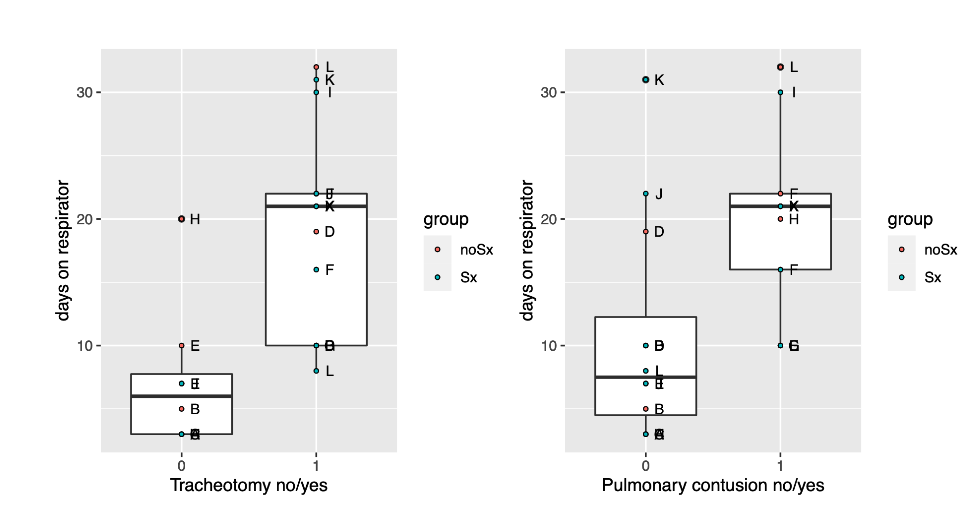


Supplementary Figure S2: Ventilator time stratified by tracheotomy and pulmonary contusion. Days on ventilator correspond to the total time for both groups.


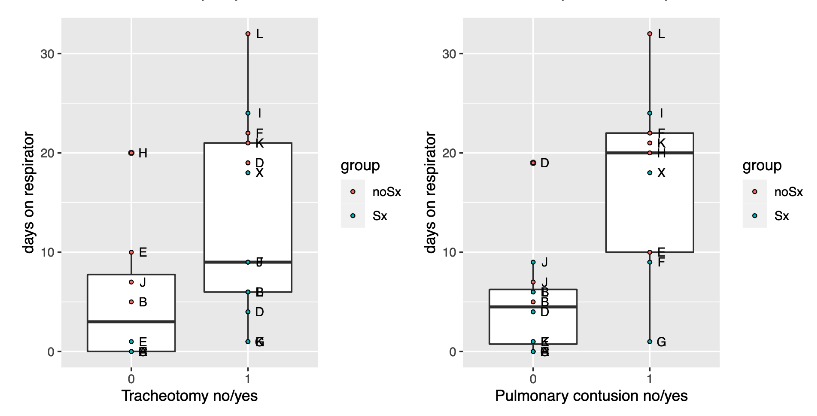


Supplementary Figure S3: Ventilator time stratified by tracheotomy and pulmonary contusion. Days on respirator corresponds to the total time for No-Sx group and time after stabilization for Sx group


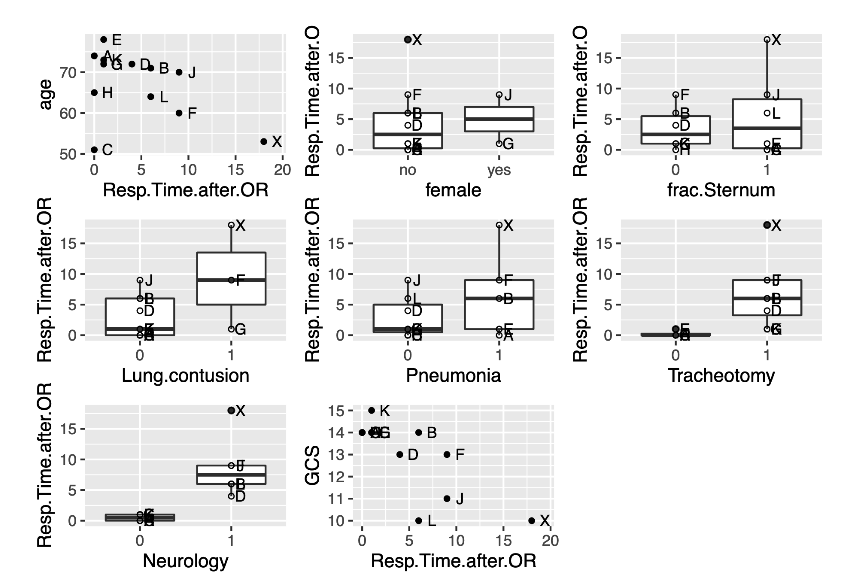


Supplementary Figure S4: Data from Sx group, patient I excluded


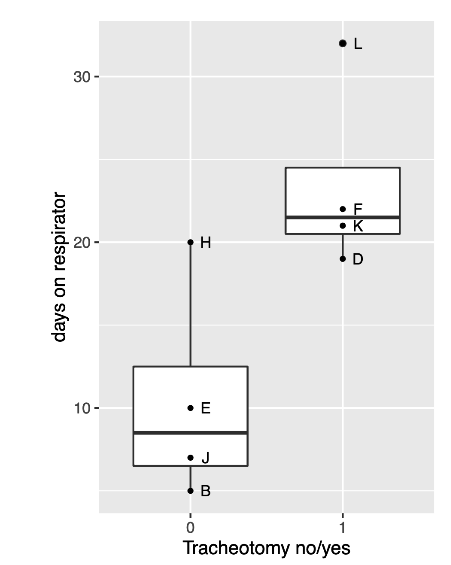


Supplementary Figure S5: Ventilator time of No-Sx group stratified by tracheotomy

Supplementary Figure S6: Visual representation of length of ventilator support of No-Sx group. Deceased patients are highlighted in purple.

Supplementary Figure S7: Visual representation of length of ventilator support of Sx group stratified according to GCS. Purple symbols denote the time point of stabilization surgery, green symbols the time point when patients were released from the ventilator. The Median length of ventilator support is 10 days.

| **Model Summary** | | | | | |
| --- | --- | --- | --- | --- | --- |
| Number of observations | Fstatistics | pvalue | R square | Adjusted R square | Residual std. error |
| 21 | 10.02 on 2 and 18 DF | 0.001187 | 0.5269 | 0.4744 | 6.859 on 18 degrees of freedom |
|  | | | | | |
| **Coefficients** | | | | | |
|  | Estimate | Std. error | t value | Pr( >\|t\| ) | 95% CI |
| Intercept | 5.569 | 2.550 | 2.184 | 0.04243 |  |
| Tracheotomy | 10.195 | 3.213 | 3.173 | 0.00527 | 3.44;16.95 |
| Pulmonary contusion | 6.724 | 3.153 | 2.132 | 0.04702 | 0.10;13.35 |
| Dependent variable: total time on ventilator | | | | | |

Supplementary Table S1

| **Model Summary** | | | | | |
| --- | --- | --- | --- | --- | --- |
| Number of observations | Fstatistics | pvalue | R square | Adjusted R square | Residual std. error |
| 21 | 14.57 on 3 and 17 DF | 5.942e-05 | 0.72 | 0.6706 | 5.499 on 17 degrees of freedom |
|  | | | | | |
| **Coefficients** | | | | | |
|  | Estimate | Std. error | t value | Pr( >\|t\| ) | 95% CI |
| Intercept | 8.540 | 2.617 | 3.264 | 0.00457 |  |
| Group | -10.039 | 2.733 | -3.674 | 0.00188 | -15.80;-4.27 |
| Tracheotomy | 7.646 | 2.708 | 2.824 | 0.01170 | 1.93;13.36 |
| Pulmonary contusion | 7.42 | 2.744 | 2.704 | 0.01505 | 1.63;13.21 |
| Dependent variable: No-Sx group: total time on ventilator, Sx group: time on ventilator after stabilization | | | | | |

Supplementary Table S2

| **Model Summary** | | | | | |
| --- | --- | --- | --- | --- | --- |
| Number of observations | Fstatistics | pvalue | R square | Adjusted R square | Residual std. error |
| 12 | 15.36 on 3 and 8 DF | 0.001105 | 0.8521 | 0.7966 | 2.451 on 8 degrees of freedom |
|  | | | | | |
| **Coefficients** | | | | | |
|  | Estimate | Std. error | t value | Pr( >\|t\| ) | 95% CI |
| Intercept | 26.0487 | 6.9299 | 3.759 | 0.00555 |  |
| GCS | -1.9950 | 0.4815 | -4.144 | 0.00324 | -3.11;-0.88 |
| Tracheotomy | 4.0403 | 1.6799 | 2.405 | 0.04283 | 0.17;7.91 |
| Pneumonia | 4.2617 | 1.4479 | 2.943 | 0.01861 | 0.92;7.60 |
| Dependent variable: time on ventilator after stabilization | | | | | |

Supplementary Table S3

| **Model Summary** | | | | | |
| --- | --- | --- | --- | --- | --- |
| Number of observations | Fstatistics | pvalue | R square | Adjusted R square | Residual std. error |
| 8 | 6.245 on 6 degrees of freedom | 0.02582 | 0.5909 | 0.5227 | 6.245 on 6 degrees of freedom |
|  | | | | | |
| **Coefficients** | | | | | |
|  | Estimate | Std. error | t value | Pr( >\|t\| ) | 95% CI |
| Intercept | 10.5 | 3.122 | 3.363 | 0.0152 |  |
| Tracheotomy | 13 | 4.416 | 2.994 | 0.0258 | 2.19;23.81 |
| Dependent variable: total time on ventilator | | | | | |

Supplementary Table S4

Supplementary Table S5: Group characteristics

Supplementary Table S6: Patient characteristics and follow up details
